# Identification and characterization of ATM founder mutation in BRCA-negative breast cancer patients of Arab ethnicity

**DOI:** 10.1038/s41598-023-48231-0

**Published:** 2023-11-27

**Authors:** Rong Bu, Abdul K. Siraj, Maha Al-Rasheed, Kaleem Iqbal, Saud Azam, Zeeshan Qadri, Wael Haqawi, Asma Tulbah, Fouad Al-Dayel, Osama Almalik, Khawla S. Al-Kuraya

**Affiliations:** 1 Human Cancer Genomic Research, Research Center, King Faisal Specialist Hospital and Research Center, 11211 Riyadh, Saudi Arabia; 2 Department of Pathology and Laboratory Medicine, King Faisal Specialist Hospital and Research Center, 11211 Riyadh, Saudi Arabia; 3 Department of Surgery, King Faisal Specialist Hospital and Research Center, 11211 Riyadh, Saudi Arabia; 4 Research Centre at KFNCCC, Human Cancer Genomic Research, King Faisal Specialist Hospital and Research Center, MBC#98-16, P.O. Box 3354, 11211 Riyadh, Saudi Arabia

**Keywords:** Breast cancer, Cancer genetics

## Abstract

Breast cancer (BC) is the most prevalent malignancy among women worldwide with germline pathogenic variants/likely pathogenic variants (PVs/LPVs) in *BRCA1/2* accounting for a large portion of hereditary cases. Recently, heterozygous PVs/LPVs in the ATM serine/threonine kinase or Ataxia-telangiectasia mutated gene (*ATM*) has been identified as a moderate susceptibility factor for BC in diverse ethnicities. However, the prevalence of *ATM* PVs/LPVs in BC susceptibility in Arab populations remains largely unexplored. This study investigated the prevalence of *ATM* PVs/LPVs among BC patients from Saudi Arabia, employing capture-sequencing technology for *ATM* PVs/LPVs screening in a cohort of 715 unselected BC patients without *BRCA1/2* PVs/LPVs. In addition, founder mutation analysis was conducted using the PHASE program. In our entire cohort, four unique PVs/LPVs in the *ATM* gene were identified in six cases (0.8%). Notably, one recurrent LPV, c.6115G > A:p.Glu2039Lys was identified in three cases, for which haplotype analysis confirmed as a novel putative founder mutation traced back to 13 generations on average. This founder mutation accounted for half of all identified mutant cases and 0.4% of total screened cases. This study further reveals a significant correlation between the presence of *ATM* mutation and family history of BC (p = 0.0127). These findings underscore an approximate 0.8% prevalence of *ATM* germline PVs/LPVs in Arab BC patients without *BRCA1/2* PVs/LPVs and suggest a founder effect of specific recurrent *ATM* mutation. These insights can help in the design of a genetic testing strategy tailored to the local population in Saudi Arabia, thereby, enabling more accurate clinical management and risk prediction.

## Introduction

Breast cancer (BC) is the most common cancer among women, accounting for a significant share of cancer-related morbidity and mortality worldwide^1–3^. BC incidence varies across ethnicities, thereby emphasizing the need for ethnic-specific genetic risk assessment. In the Middle Eastern population, including Saudi Arabia, BC is the most prevalent malignancy among women^4–6^. Notably, BC appears to manifest at an earlier age and presented with advanced stage in these populations compared to their western counterparts, indicating unique genetic predisposition factors^7–11^.

Among genetic factors, germline pathogenic variants/likely pathogenic variants (PVs/LPVs) in the *BRCA1* and *BRCA2* genes account for a large proportion of hereditary breast cancer cases worldwide^12,13^. Previously, the prevalence of *BRCA* mutation in Middle Eastern BC patients has been estimated to be 3.4% in overall cases. On the other hand, high-risk BC patients (positive family history, early onset, TNBC, and cases with bilateral breast cancer) demonstrated that 6.4% had *BRCA* mutation^14^. However, beyond *BRCA1/2*, other genes are being recognized as contributors to BC predisposition, with the *ATM* serine/threonine kinase or Ataxia-telangiectasia mutated gene (*ATM*) emerging as a notable moderate susceptibility gene^15–19^. *ATM* gene plays a crucial role in DNA damage response; cell cycle control as well as telomere maintenance and heterozygous PVs/LPVs of *ATM* have two to 13- fold-increased risk of BC development^20–23^. However, the distribution and the contribution of *ATM* PVs/LPVs to BC susceptibility in Arab populations remains limited.

Arab genetic architecture is very distinctive with known genetic drift among their population^24^. The genetic drift and the ensuing founder effect represent significant genetic forces that can shape the genetic landscape of population, contributing to a unique spectrum of disease-causing variants. This phenomenon is particularly evident in populations with high levels of consanguinity such as in the Middle Eastern Arab population^25,26^. Genetic drift, alongside the population-specific selection pressures and environmental exposures, can result in the enrichment of specific disease-associated PVs/LPVs including those in cancer predisposition genes. Hence, the identification and characterization of these PVs/LPVs and founder mutations can provide valuable insights into the unique genetic underpinnings of BC in the Arab population.

Therefore, we conducted this study to determine the prevalence, spectrum, and founder effect of *ATM* germline PVs/LPVs in a large cohort of *BRCA1/2* negative BC patients from Saudi Arabia. Such knowledge could contribute to enhancing personalized management strategies for BC patients in the region.

## Methods

### Sample selection

A total of 715 *BRCA* PVs/LPVs negative BC cases were included in this study. All these patients were diagnosed and treated at King Faisal Specialist Hospital & Research Centre (KFSH&RC). All clinicopathological data were collected from case records and presented in Table 1. The eighth edition of the American Joint Committee on Cancer (AJCC) staging system was utilized to determine the stage of breast cancer^27^. The Institutional Review Board of the King Faisal Specialist Hospital & Research Center approved this study. Since only archival tissue specimens and retrospective patient data were utilized, the Research Advisory Council (RAC) provided a waiver of consent under project RAC # 2140 008.

**Table 1:** Clinico-pathological characteristics of breast cancer patients included in the study (n = 715).

Clinico-pathological variables	n (%)
Age at diagnosis, years	
Mean ± SD	41.2 ± 9.9
Median (range)	39.4 (13–84)
≤ 50	614 (85.9)
> 50	101 (14.1)
Family history of breast cancer	
No	582 (81.4)
Yes	133 (18.6)
Bilateral breast cancer	
Yes	12 (1.7)
No	703 (98.3)
Lymph node status	
Negative	260 (36.4)
Positive	434 (60.7)
Unknown	21 (2.9)
Distant metastasis	
Absent	628 (87.9)
Present	66 (9.2)
Unknown	21 (2.9)
Stage	
I	98 (13.7)
II	285 (39.9)
III	245 (34.3)
IV	66 (9.2)
Unknown	21 (2.9)
Histologic grade	
Well differentiated	48 (6.7)
Moderately differentiated	322 (45.0)
Poorly differentiated	310 (43.4)
Unknown	35 (4.9)
Estrogen receptor status	
Positive	393 (55.0)
Negative	291 (40.7)
Unknown	31 (4.3)
Progesterone receptor status	
Positive	360 (50.3)
Negative	323 (45.2)
Unknown	32 (4.5)
Her-2 neu status	
Positive	233 (32.6)
Negative	441 (61.7)
Unknown	41 (5.7)

### DNA extraction

In our study, Gentra DNA Isolation Kit (Gentra, Minneapolis, MN, USA) was utilized to extract DNA samples from normal formalin-fixed and paraffin-embedded (FFPE) breast cancer or ovarian cancer tissue following the manufacturer's protocols as described in our previous study^28^. Two pathologists examined the histopathology slides to ensure that normal tissues were obtained from different FFPE blocks such as uninvolved lymph nodes or non-cancerous breast tissue away from the tumor in order to minimize somatic contamination.

### Capture sequencing analysis

A custom-designed gene panel was used to perform Targeted capture sequencing on 715 samples^29^. The DNA samples with A260/A280 ratio between 1.8 and 2.0 were selected for library construction. The preparation of the sequencing library was carried out by randomly fragmenting the DNA sequences as described in the previous study^30^.The BCL (base calls) produced by Illumina HiSeq 4000 platform were transformed into FASTQ files through the bcl2fastq software (v2.16). Subsequently, the sequence reads in FASTQ format from each sample were aligned to the reference human genome (GRCh37/hg19) using Burrows-Wheeler aligner (BWA)^31^. We generated BAM files, addressed PCR duplicates, and conducted local realignment using a combination of Picard-tools and Genome Analysis Toolkit (GATK) as described in a previous study^32^.

### Variant calling

GATK was employed for variant calling, followed by the annotation of the variants using ANNOVAR^33^. Annotations were sourced from databases including dbSNP138, 1000 Genomes, ESP6500, Exome Aggregation Consortium (ExAC), ClinVar and other relevant genome databases. The classification of pathogenic and likely pathogenic variants adhered to the recommended guidelines established by the American College of Medical Genetics and Genomics and the Association of Molecular Pathology (ACMG/AMP)^34^.

### Haplotype analysis

Genotyping was performed on custom designed High-throughput Illumina Infinium SNP Genotyping Array with 778,783 SNPs following manufacturer’s instruction (Illumina Inc). Normalized signal intensity and genotype were computed using Illumina Bead Array Files Python library. Quality checking was done by plotting p10 GC and sample call rate and a text file containing the genotype of entire samples and probes was generated. SNP data for 100 controls was also included from our in-house database.

Haplotype construction was performed on two samples and 100 controls utilizing PHASE version 2.1.1 algorithm^35,36^. Count of variant positions, nucleotide positions of variants and sample genotypes for each sample and controls at those positions were provided as an input in the algorithm. Following parameter were set: number of iterations = 100, thinning interval = 1, burn-in = 100. DMLE + version 2.3^37^, which is a linkage disequilibrium mapping software, was utilized to estimate the age of variants with founder effect. This software uses Markov Chain Monte Carlo algorithm for Bayesian estimation of mutation age as described previously^38^. The analysis was performed as described in our previous study^39^.

### Statistical analysis

Contingency table analysis and Fisher’s exact tests were used to analyze the association clinico-pathological variables and *ATM* mutations. Two-sided tests employed for analyses with a significance threshold set at a *p*-value < 0.05. All data analyses were carried out using the JMP14.0 software package developed by SAS Institute, Inc., Cary, NC.

### Ethics approval and consent to participate

The Institutional Review Board of the King Faisal Specialist Hospital and Research Center approved this study and since only archival tissue specimens and retrospective patient data were used, the Research Advisory Council (RAC) of King Faisal Specialist Hospital and Research Center provided waiver of consent under project RAC # 2140 008.

## Results

### Identification of PVs/LPVs in ATM gene and founder mutation analysis

In entire cohort, four unique PVs/LPVs in *ATM* gene were identified in six cases accounting for 0.8% of all cases. Among these four variants, one recurrent missense LPV was detected in three cases, accounting for 50% of all mutant cases and 0.4% of all sequenced cases. Other three were pathogenic variants including two splicing PVs and one frameshift PV, each one observed in one case, accounting for 0.1% of all cases (Table 2). All these variants were reported previously. Interestingly, four out of six mutant cases were reported to have positive family history of BC, accounting for 3% (4/133) of all family history positive cases (Table 3).

**Table 2:** *ATM* PVs/LPVs identified in our cohort.

Chr	Position	Ref	Alt	HGVS	Mutation type	Pathogenicity	# of cases	dbSNP ID
chr11	108098502	G	T	c.73-1G > T	Splicing	Likely pathogenic	1	rs1555054043
chr11	108106446	A	–	c.381delA:p.Thr127fs	Frameshift	Pathogenic	1	rs587781831
chr11	108186757	G	A	c.6115G > A:p.Glu2039Lys	Missense	Likely pathogenic	3	rs864622251
chr11	108206571	G	A	c.8152-1G > A	Splicing	Pathogenic	1	rs1398616877

**Table 3:** Clinico-pathological characteristics of ATM mutant cases in breast cancer (n = 6).

S no	Age	Family history	Tumor laterality	pT	pN	pM	Stage	Grade	Status	ER	PR	Her-2	TNBC	Mutation	dbSNP ID
1	63	YES (first degree relative—breast cancer)	Unilateral	T3	N2	M1	IV	G2	Dead (disease progression)	Negative	Negative	Negative	Yes	c.6115G > A: p.Glu2039Lys	rs864622251
2	32	No	Unilateral	T3	N0	M0	II	G2	Alive	Positive	Positive	Positive	No	c.6115G > A: p.Glu2039Lys	rs864622251
3	39	No	Unilateral	T3	N0	M0	II	G2	Alive	Positive	Positive	Positive	No	c.6115G > A: p.Glu2039Lys	rs864622251
4	39	YES (first degree relative—breast cancer)	Unilateral	T2	N1	M0	II	G2	Alive	Positive	Positive	Negative	No	c.73-1G > T	rs1555054043
5	38	YES (first degree relative—breast cancer)	Unilateral	T4	N1	M0	III	G3	Alive	Positive	Negative	Negative	No	c.381delA: p.Thr127fs	rs587781831
6	59	YES (second degree relative—breast cancer)	Unilateral	T3	N1	M0	III	G2	Alive	Positive	Positive	Negative	No	c.8152-1G > A	rs1398616877

Haplotype construction was performed for two cases with recurrent variant and sufficient DNA sample utilizing PHASE version 2.1.1 algorithm. Our results revealed that the two carriers of c.6115G > A: p.Glu2039Lys in *ATM* gene shared the same haplotype with length of ~ 1.4 MB (Supplementary Table 1), suggesting that this recurrent mutation is putative novel founder mutation derived from common ancestor. Furthermore, the result of age estimation showed the average age of this founder mutation as 13 generations (10–17 generations; 95% CI).

### Clinico-pathological characteristics of BC patients with ATM PV/LPVs

Median age of the *ATM* mutant cases was 39 years (range = 32–63 years). All the six *ATM* mutant cases were unilateral tumors. Majority of the *ATM* mutant cases were larger tumors (T3/T4–83.3%; 5/6), had lymph node metastasis (66.7%; 4/6), were advanced stage (Stage III/IV–50%; 3/6) and hormone receptor positive (83.3%; 5/6). Of the six *ATM* mutant cases, one patient died due to disease progression. Interestingly, we found a significant association between *ATM* mutations and family history of breast cancer (*p* = 0.0127), with three patients having a first-degree relative and one patient having a second-degree relative being diagnosed with BC (Table 3). However, no significant association was noted between ATM mutation and age of onset (early-onset vs. late-onset) of BC, lymph node status, tumor stage, grade, estrogen receptor, progesterone receptor and *Her-2* status (Table 4).

**Table 4:** Clinico-pathological associations of *ATM* mutant breast cancer patients.

Clinico-pathological variables	*ATM* mutant (n = 6)	*ATM* wildtype (n = 709)	p value
	n (%)	n (%)	
Age at diagnosis, years			
Mean ± SD	45 ± 12.7	41.2 ± 9.8	
Median (range)	39 (32 – 63)	39.7 (13 – 84)	
≤ 50	4 (66.7)	610 (86.0)	0.2027
> 50	2 (33.3)	99 (14.0)	
Family history of breast cancer			
No	2 (33.3)	580 (81.8)	0.0127
Yes	4 (66.7)	129 (18.2)	
Bilateral breast cancer			
Yes	0	12 (1.7)	1.0000
No	6 (100.0)	697 (98.3)	
Lymph node status			
Negative	2 (33.3)	258 (37.5)	1.0000
Positive	4 (66.7)	430 (62.5)	
Distant metastasis			
Absent	5 (83.3)	623 (90.5)	0.4522
Present	1 (16.7)	65 (9.5)	
Stage			
I	0	98 (14.2)	0.7233
II	3 (20.0)	282 (41.0)	
III	2 (33.3)	243 (35.3)	
IV	1 (16.7)	65 (9.5)	
Histologic grade			
Well differentiated	0	48 (7.1)	0.2026
Moderately differentiated	5 (83.3)	317 (47.0)	
Poorly differentiated	1 (16.7)	309 (45.9)	
Estrogen receptor status			
Positive	5 (83.3)	388 (57.2)	0.2479
Negative	1 (16.7)	290 (42.8)	
Progesterone receptor status			
Positive	4 (66.7)	356 (52.6)	0.6889
Negative	2 (33.3)	321 (47.4)	
Her-2 neu status			
Positive	2 (33.3)	231 (34.6)	1.0000
Negative	4 (66.7)	437 (65.4)	

## Discussion

Our findings underscore the pivotal role of *ATM* PVs/LPVs in the predisposition of BC in Arab populations, a group traditionally under presented in BC genomics. This study uncovered a recurrent *ATM* LPV, c.6115G > A:p.Glu2039Lys, identified as novel putative founder mutation within Arab populations. The observation of this recurrent variant draws attention to the specific genetic landscape in this population and emphasizes the unique contribution of *ATM* PV/LPV to the BC risk.

In our study, 0.8% of 715 unselected Saudi Arabian patients with *BRCA* mutation-negative BC carried *ATM* germline PVs/LPVs. *BRCA1/2* are well known hereditary breast cancer predisposition genes and have been extensively studied. However, it is now estimated that more than one-half of individuals with a pathogenic variant (PV) who meet the National Comprehensive Cancer Network (NCCN) testing criteria for hereditary breast and ovarian cancer (HBOC) carry PVs in genes other than *BRCA1* or *BRCA2*^40^. According to previous reports, 3.4% of Middle Eastern breast cancer cases carry *BRCA1/2* mutation^14^. However, genetic basis for a large proportion of BC patients is still unknown, therefore, it is crucial to investigate the prevalence of other genes in breast cancer cases to facilitate the development of a genetically-tailored testing strategy for the indigenous population of Saudi Arabia, ultimately enhancing the precision of clinical management and risk prediction. The study further reveals a significant correlation between the presence of *ATM* PV/LPV and family history of breast cancer. Majority of *ATM* PVs/LPVs carries have ER and/or PR-positive breast cancer or large tumors.

The observed prevalence of *ATM* PVs/LPVs in our cohort (0.8%) aligns with frequencies reported in other population, reaffirming *ATM* as a moderate-risk BC susceptibility gene globally. Based on previous reports, *ATM* mutation frequency ranges from 0.5 to 4%, depending on the population studied^41–46^

In comprehensive sequencing study of BC cases and controls, *ATM* was identified as one of the several genes with mutations significantly associated with BC risk^41^. This study reported a similar frequency of *ATM* PVs/LPVs (0.85%) among BC cases. Another large case–control study found that rare *ATM* PV/LPV prevalence of 0.4% in Chinese BC patients^47^. Two previous large-scale gene panel studies in Caucasian BC patients found that the prevalence of *ATM* PVs/LPVs was approximately 1%^40,48^.

Remarkably, certain populations have exhibited a higher prevalence of *ATM* PVs/LPVs. A study from Netherlands has reported a considerably higher frequency of *ATM* mutation (~ 4%)^44^. Similarly, a study investigating Irish individuals reported a prevalence of *ATM* mutations of 2.9%^45^. Another study based on Spanish population also identified 1.9% frequency of *ATM* mutation among their BC patients^46^.

These differences underscore the variable contributions of *ATM* mutations across different ethnicities, potentially reflecting distinct founder effect. The significant association of *ATM* mutations with a family history of BC in our study aligns with previous reports^49,50^. BC patients with family history have been found to carry *ATM* PVs/LPVs at higher rates, further supporting its role as a hereditary BC susceptibility gene^16,22,50^. Notably, the frequency and spectrum of *ATM* mutations can be influenced by genetic drift, population-specific factors, and environmental exposure, warranting further investigation. Interestingly, if we restrict the prevalence of *ATM* PVs/LPVs among BC patient with positive family history, the frequency will rise to 3% (4/133). Therefore, the family history of breast cancer should be taken into account during genetic counselling.

Similar to patients with any high to moderate risk of BC, surveillance is necessary for patients with *ATM* PVs/LPVs and their relatives. It was suggested for women with family history of breast to undergo early screening by mammogram and MRI^51^. According to recent guidelines, women who are carrier for *ATM* germline PVs/LPVs should go under surveillance by at least age of 40 years since their lifetime risk of BC is likely higher than 25%. Large-scale, age-matched case–control studies are vital to investigate the lifetime risk of BC in carriers of the *ATM* PVs/LPVs in the Arab population.

Our study findings hold significant clinical implications, besides enhancing our understanding of BC genomic landscape in Arab population, enabling a more inclusive and precise approach to risk assessment, genetic testing and patient management in this population. With the advent of precision medicine, incorporation of *ATM* genetic testing into standard BC risk assessment, particularly in population where *ATM* PVs/LPVs are prevalent, can offer more personalized therapeutics and preventive strategies.

In conclusion, our study showed that the prevalence of *ATM* germline PVs/LPVs in *BRCA* mutation-negative patients in Arab population was approximately 0.8%. Our findings suggest founder effect for specific recurrent *ATM* PV/LPV, providing vital insights into the genomics of BC in Arab population. This data could be used to shape a genetic testing strategy customized for Arab population, thereby enabling more accurate clinical management, including risk prediction, surveillance, prevention and treatment of BC.

### Supplementary Information

Supplementary Table 1.

## Data Availability

All data generated or analyzed during this study are included in this published article and its Supplementary Information files.
