# Supplementary material for: Identification and characterization of ATM founder mutation in BRCA-negative breast cancer patients of Arab ethnicity
Source: Sci Rep. 2023 Nov 27;13:20924. doi: 10.1038/s41598-023-48231-0 (PMC10684510; doi:10.1038/s41598-023-48231-0)
Supplement: Supplementary file 1 — Supplementary Table 1. [file 41598_2023_48231_MOESM1_ESM.docx]

**Supplementary Table 1: Shared haplotype in Breast cancer cases with *ATM* founder mutation.**

| **Position** | 107037976 | 107130964 | 107134481 | 107140392 | 107140595 | 107286943 | 107297902 | 107299623 | 107353313 | 107355600 | 107448756 | 107481518 | 107494405 | 107496966 | 107503447 | 107567288 | 107571850 | 107584654 | 107586096 | 107595881 | 107598176 | 107614258 | 107621538 | 107627444 | 107627745 | 107644296 | 107655949 | 107693120 | 107740817 | 107772104 | 107807744 | 107833545 | 107834123 | 107841291 | 107843602 | 107874113 | 107884273 | 107885991 | 107918899 | 108032707 | 108040484 | 108068954 | 108115587 | 108138003 | 108159732 | 108175462 | 108186757 | 108339044 | 108383676 | 108384661 | 108385251 | 108409442 | 108412369 | 108440814 | 108452651 | 108464209 | 108468768 | 108495541 | 108498885 | 108535716 | 108599993 | 108846480 |
| --- | --- | --- | --- | --- | --- | --- | --- | --- | --- | --- | --- | --- | --- | --- | --- | --- | --- | --- | --- | --- | --- | --- | --- | --- | --- | --- | --- | --- | --- | --- | --- | --- | --- | --- | --- | --- | --- | --- | --- | --- | --- | --- | --- | --- | --- | --- | --- | --- | --- | --- | --- | --- | --- | --- | --- | --- | --- | --- | --- | --- | --- | --- |
| **dbSNP RS ID** | rs10789572 | rs12419678 | rs10789589 | rs7946836 | rs7111266 | rs17106909 | rs17666075 | rs35968518 | rs17107015 | rs510366 | rs1943684 | rs10431058 | rs12576509 | rs12226290 | rs10789626 | rs11212330 | rs683266 | rs17670446 | rs12794422 | rs1384010 | rs7927287 | rs1351518 | rs520906 | rs12802219 | rs12802831 | rs632411 | rs607506 | rs1963626 | rs11212413 | rs563811 | rs11601743 | rs4754282 | rs10789646 | rs4028250 | rs1504747 | rs12577263 | rs17616401 | rs12418162 | rs11212495 | rs35303872 | rs34052882 | rs683312 | rs3218674 | rs1800056 | rs3092856 | rs1801516 | **ATM Mutation** | rs17618313 | rs10749920 | rs12146448 | rs11212684 | rs2640784 | rs12804124 | rs2852199 | rs7105022 | rs2640738 | rs9666351 | rs7937558 | rs10160242 | rs3809032 | rs12793497 | rs6589039 |
| **Sample1** | A | A | **B** | A | A | B | B | A | A | B | A | A | B | A | A | A | B | B | B | B | A | A | A | B | B | B | B | A | B | B | A | A | B | B | B | B | A | B | A | A | B | B | B | A | B | B |  | B | A | B | B | A | A | B | B | B | A | B | **A** | B | B | B |
| **Sample2** | B | A | **A** | A | A | B | B | A | A | B | A | A | B | A | A | A | B | B | B | B | A | A | A | B | B | B | B | A | B | B | A | A | B | B | B | B | A | B | A | A | B | B | B | A | B | B |  | B | A | B | B | A | A | B | B | B | A | B | **B** | B | B | A |
